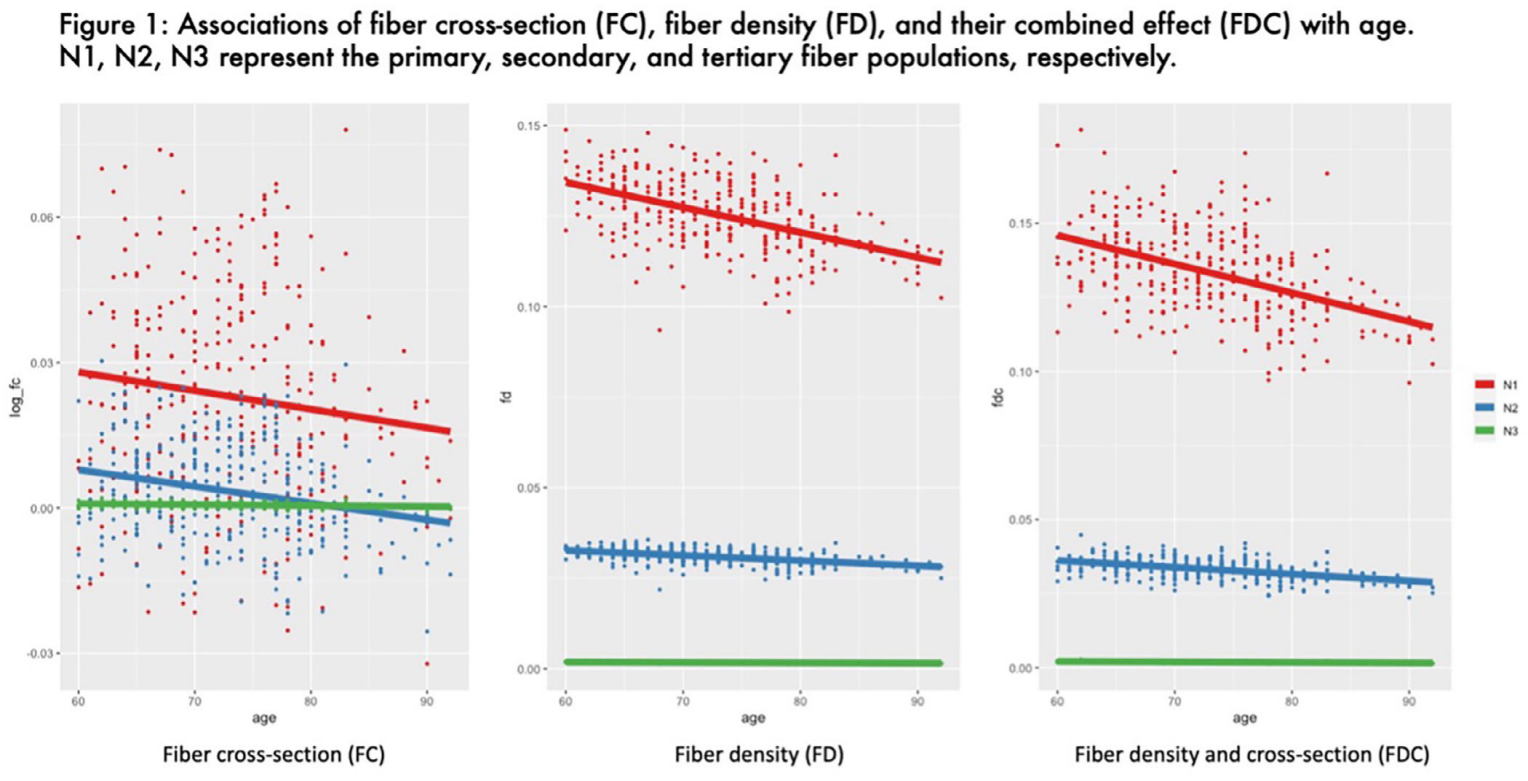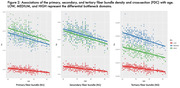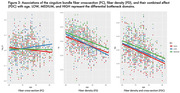# Using fixel‐based analysis to understand the role of crossing fibers in age‐related white matter decline

**DOI:** 10.1002/alz.090876

**Published:** 2025-01-09

**Authors:** Aditi Sathe, Niranjana Shashikumar, Elizabeth E. Moore, Kimberly R. Pechman, Kurt Schilling, Bennett A. Landman, Katherine A. Gifford, Andrew R. Bender, Timothy J. Hohman, Angela L. Jefferson, Derek B. Archer

**Affiliations:** ^1^ Vanderbilt Memory & Alzheimer’s Center, Vanderbilt University Medical Center, Nashville, TN USA; ^2^ Vanderbilt University Institute of Imaging Science, Vanderbilt University Medical Center, Nashville, TN USA; ^3^ Department of Radiology & Radiological Sciences, Vanderbilt University Medical Center, Nashville, TN USA; ^4^ Department of Electrical and Computer Engineering, Vanderbilt University, Nashville, TN USA; ^5^ Department of Biomedical Engineering, Vanderbilt University, Nashville, TN USA; ^6^ Vanderbilt University Institute on Imaging Science, Vanderbilt University Medical Center, Nashville, TN USA; ^7^ Department of Neurology, Vanderbilt University Medical Center, Nashville, TN USA; ^8^ Cleveland Clinic Lou Ruvo Center for Brain Health, Las Vegas, NV USA; ^9^ Vanderbilt Genetics Institute, Vanderbilt University Medical Center, Nashville, TN USA; ^10^ Vanderbilt Memory & Alzheimer’s Center, Department of Neurology, Vanderbilt University Medical Center, Nashville, TN USA; ^11^ Department of Medicine, Vanderbilt University Medical Center, Nashville, TN USA; ^12^ Vanderbilt Memory and Alzheimer’s Center, Vanderbilt University Medical Center, Nashville, TN USA; ^13^ Vanderbilt Genetics Institute, Institute for Medicine and Public Health Vanderbilt University Medical Center, Nashville, TN USA

## Abstract

**Background:**

Aging is linked to significant white matter abnormalities, which are often studied using traditional diffusion tensor imaging (DTI) metrics; however, these traditional metrics have limited sensitivity/specificity to neurobiological characteristics. Here, we use fixel‐based analysis (FBA) – an approach with more precision in areas of crossing fibers – to study age‐related white matter microstructural decline.

**Method:**

This study uses cross‐sectional data from the Vanderbilt Memory & Aging Project cohort [n=325, age at baseline: 72.9 ± 7.3 years, 40% mild cognitive impairment (MCI)]. Diffusion MRI was preprocessed using the PreQual pipeline and MRtrix was used to calculate fiber density (FD), fiber‐bundle cross‐section (FC), their combined effect (FDC), and complexity (CX) of the multi‐fiber organization within a voxel. To account for heterogeneity in the number of crossing fibers, the TractSeg Bottleneck atlas was split into distinct low/medium/high regions‐of‐interest (ROIs) based on the number of white matter bundles converging within a single voxel. Fixel‐based metrics were then quantified in each ROI. We conducted a linear regression to determine the association between age and all FBA metrics, covarying for age, sex, education, race/ethnicity, apolipoprotein‐e4 status, and cognitive status.

**Result:**

FD, FC, and FDC all show significant decline with age, with the primary fiber bundle displaying the greatest reductions in FDC (p<0.0001). This decrease is predominantly driven by changes in FD, while FC remains relatively consistent over time. Alterations to the primary fiber bundle are the most prominent in the high and medium bottleneck ROIs as opposed to low (p<0.0001). FBA of the cingulum bundle (a tract vulnerable to abnormal aging) also showed differential decline in FD and FC over time. Figures 1‐3 provide illustrations of notable findings. The low/medium/high bottleneck ROIs correlate exceedingly well with global CX, supporting the use of the Bottleneck atlas to interpret structural white matter differences.

**Conclusion:**

This study provides novel insights into the micro‐ and macro‐structural differences underlying age‐related white matter alterations. The findings underscore the value of FBA in characterizing age‐related white matter degeneration, particularly in regions susceptible to bottleneck effects. This research also lays the foundation for future investigations into tract‐specific patterns of neurodegeneration in aging.